# d-Aspartate consumption selectively promotes intermediate-term spatial memory and the expression of hippocampal NMDA receptor subunits

**DOI:** 10.1038/s41598-021-85360-w

**Published:** 2021-03-17

**Authors:** Gergely Zachar, Róbert Kemecsei, Szilvia Márta Papp, Katalin Wéber, Tamás Kisparti, Teadora Tyler, Gábor Gáspár, Tamás Balázsa, András Csillag

**Affiliations:** Department of Anatomy, Histology and Embryology, Faculty of Medicine, Semmelweis University, 58 Tűzoltó u, Budapest, 1094 Hungary

**Keywords:** Hippocampus, Spatial memory

## Abstract

d-Aspartate (d-Asp) and d-serine (d-Ser) have been proposed to promote early-phase LTP in vitro and to enhance spatial memory in vivo. Here, we investigated the behavioural effects of chronic consumption of d-Asp and d-Ser on spatial learning of mice together with the expression of NMDA receptors. We also studied the alterations of neurogenesis by morphometric analysis of bromo-deoxyuridine incorporating and doublecortin expressing cells in the hippocampus. Our results specify a time period (3–4 h post-training), within which the animals exposed to d-Asp (but not d-Ser) show a more stable memory during retrieval. The cognitive improvement is due to elimination of transient bouts of destabilization and reconsolidation of memory, rather than to enhanced acquisition. d-Asp also protracted reversal learning probably due to reduced plasticity. Expression of GluN1 and GluN2A subunits was elevated in the hippocampus of d-Asp (but not d-Ser) treated mice. d-Asp or d-Ser did not alter the proliferation of neuronal progenitor cells in the hippocampus. The observed learning-related changes evoked by d-Asp are unlikely to be due to enhanced proliferation and recruitment of new neurones. Rather, they are likely associated with an upregulation of NMDA receptors, as well as a reorganization of receptor subunit assemblies in existing hippocampal/dentate neurons.

## Introduction

Though similar in physical and chemical properties to their chiral l-enantiomers, d-amino acids (DAAs) do not form regular building blocks of proteins^1^. Instead, they are known to occur in a free form, functioning as bioregulators and transmitters^2^. They are thought to be involved in the regulation of hormone synthesis during ontogeny and in neurotransmission in the CNS^3^. Of the DAAs, d-serine (d-Ser) and d-aspartate (d-Asp) have been found to show the highest concentration in mammals^4^.

DAAs can be ingested from food and drink via absorption from the intestinal tract^5,6^, and can pass the blood–brain barrier^7^, also reaching the hippocampus, causing functional connectivity changes^8^. By overriding the postembryonic decline, chronic consumption of a d-amino acid enriched diet is known to lead to persistent increase (2–fivefold) of d-Asp in the brain tissue^5,9^, and to an elevated concentration of d-Asp also in the extracellular fluid^9^. In a previous study, based on in vitro microdialysis and tissue extracts in the striatum of 2-day-old domestic chicks, we found that a surprisingly high proportion (ca. 37%) of extracellular aspartate (released) was in the D form, whilst d-Asp constituted only about 1% of total aspartate in the tissue^10^. Notably, this result was obtained at an early developmental stage (under conditions of high d-Asp), *i.e.* similar to the situation of the present study on adult mice, where high d-Asp level was obtained by the consumption of d-Asp as a dietary supplement. It also indicates the potential importance of d-Asp co-acting with l-Asp on N-methyl-d-aspartate receptors (NMDARs).

Since the concentration of DAAs is high in embryonic and early postembryonic brain but it decreases rapidly thereafter^11–13^, they might promote cell proliferation in the adult brain as well. Albeit the synthetic enzyme of d-Asp has not been unequivocally established, the degrading enzyme d-aspartate oxidase (DDO) has been well known for some time^14,15^. An increase of d-Asp – suggesting ongoing synthesis – was demonstrated in postnatal adult DDO KO mice^16^.

d-Asp binds to the glutamate binding subunit of NMDAR as an agonist either directly^17^, or through enzymatic transformation into NMDA^18^. Since d-Asp directly acts on NMDARs, it is plausible to assume that d-Asp might influence behavioural plasticity via modification of NMDAR expression and subunit composition. Due to a developmental switch^19,20^, the heterotetrameric NMDARs containing GluN1/GluN2B subunits get gradually replaced by those composed of GluN1/GluN2A subunits, or other triheteromeric forms. Any potential learning-related effects of d-Asp have to be discussed against this backdrop. d-Asp and its derivative NMDA are potent agonists of ionotropic NMDA receptors, and the binding site is the GluN2 subunit^21^, similarly to l-Glu and l-Asp, evoking a transient rise of intracellular Ca^2+^ ^22^. Notably, an effect of d-Asp on AMPA receptors has also been reported^23,24^. Apart from immediate action on the receptor and its consequences in neuronal metabolism, d-Asp may also bring about alterations of receptor expression and internalization, particularly in case of chronic administration (such as in the present study).

Both the GluN2A and GluN2B subunit participate in the formation of long-term potentiation (LTP). The latter has specific significance due partly to the higher fractional Ca^2+^ current passed by NMDARs containing the GluN2B^25^, and partly due to their intimate link to the calcium/calmodulin-dependent protein kinase II (CaMK-II)^26^. NMDAR dependent LTP induction tends to recruit more GluN2B subunits in mossy fibre – CA3 synapses, whereas in other hippocampal fields (CA1) the activity-driven induction and insertion of GluN2A subunits is more prominent. As a result of the changes in subunit composition, the decay kinetics of the NMDA-EPSC will slow down^27^. As recently demonstrated in adult-born hippocampal dentate neurons, the GluN2A (rather than the GluN2B) subunit is more directly associated with the reversal of LTP, *i.e.* a forgetting-related mechanism^28^.

Many have suggested that the developmental role of d-Asp is associated with an increase of neurogenesis^13,16,29^, including highly specific alterations such as the proliferation of parvalbumin containing neurones^30^. Yet there is no convincing evidence to suggest that d-Asp promotes the proliferation or the survival of neurones during adulthood^31,32^. It is tempting to speculate that an increased neurogenesis in the hippocampus might contribute to the enhancement of spatial cognitive abilities^33^.

Both d-Asp and d-Ser have been proposed to facilitate cognitive processes such as learning and memory^5,6,34^. During young adulthood the upregulation of cerebral d-Asp level is always associated with increased NMDAR-dependent functions, including the enhancement of hippocampal LTP ^17,22,35^. Elevated d-Asp is known to promote both early-phase and late-phase LTP (E-LTP and L-LTP, respectively) in the CA1 area of hippocampus^17^. Both *Ddo* gene ablation and chronic d-Asp treatment are associated with increased dendritic length and spine density in pyramidal neurons of the hippocampus and PFC^35^, and with improved NMDAR-dependent spatial memory^12,22,28,29^, also counteracting the age-dependent decline^21^. However, the findings concerning the effects on spatial learning have been controversial. Several studies showed a cognitive rescuing effect of d-Asp in various rodent disease models, with notable differences^36^. More specifically, rats clearly react to chronic d-Asp consumption with improved cognitive abilities if measured by MWM^6^. However, healthy adult mice show very little – if any – cognitive improvement either after chronic consumption of d-Asp^5,21^, or by life-long exposure to elevated d-Asp due to genetic alterations^5,17,21^. More surprisingly still, in a recent report, a reduction of embryonic d-Asp (due to genetically *enhanced* DDO activity) was accompanied by cognitive improvement^30^. Therefore, our first goal was attempting to settle this issue at the phenomenological (behavioural) level, by refined analysis of spatial learning, using MWM in mouse. Once the cognitive alterations evoked by d-Asp have clearly been confirmed, we went on to investigate the underlying mechanisms in two, previously suggested, directions: the role of NMDARs and the involvement of neurogenesis in the hippocampus.

Alongside with d-Asp, the effects of the other major DAA (d-Ser) were also investigated in the present study. d-Ser is known to be expressed in the brain^37^, its distribution is correlated with that of NMDARs^12^ and biodegraded by DAA oxidase (DAO)^38^. In ontogeny, the greatest concentration of d-Ser was found during the first three postnatal weeks^39^. The main cellular source of d-Ser are type II astrocytes, implicated in synaptic strength^40^. d-Ser is transferred to synapses via the alanine-serine-cysteine-1 transport (Asc-1) pathway^41^. Once at the synapse, it acts as an agonist of the glycine binding site (GluN1 subunit) of NMDARs^42^, and likely participates in the adjustment of the sensitivity of NMDARs – thereby regulating neuronal excitability^43^. Now known as a gliotransmitter, d-Ser is thought to affect dendritic morphology and synaptic plasticity and as such it has been implicated in the aetiology of schizophrenia^44^. d-Ser is thought to play a role in the regulation of the sensitivity of NMDAR, hippocampal LTD^45–47^, and learning-related effects of acute or chronic administration of d-Ser have also been reported^34,46^. An increased proliferation of DG granule cells by d-Ser was reported in adult mice^48^.

Based on the above considerations, the following questions were investigated: (i) Is there a distinct link between the chronic consumption of d-amino acids and cognitive capabilities, in particular, in the formation of spatial memory of mice? (ii) If so, are such cognitive effects accompanied by altered expression of NMDARs? (iii) Can any cognitive effects be attributed to increased neurogenesis in the adult hippocampus?

## Materials and methods

### Experimental animals, feeding protocol

**Figure 1:**
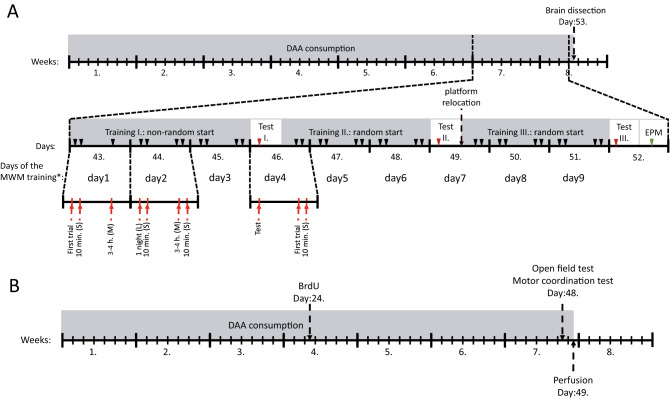
Graphical illustration of the experimental timeline of two cohorts of mice: (**A**) In Experiment 1, spatial learning ability of the mice was assessed using MWM test from day 43 to day 51. Black arrowheads denote the training trials while red arrowheads show the test trials (where the platform was not present). Red arrows represent the time position of trials within a day, along with the preceding intertrial intervals (trial types: M, L or S). *EPM* elevated plus maze test, *days of the MWM training as shown on Fig. 2. (**B**) In Experiment 2, BrdU was injected on day 24 of DAA consumption to label proliferating neurones. One day before perfusion open field test followed by a motor coordination test (rod grip test) were carried out on all experimental groups.

A total of 72 male mice of the C57BL/6 strain (in two cohorts of 31 and 41) were obtained from the laboratory animal breeding facility of the Institute of Oncology, Budapest. The animals were 6–10 weeks old at the beginning of the experiments. In the upcoming 7-week period the animals consumed one of 3 different amino acids. The animals were randomly assigned to 3 groups receiving a certain type of amino acid diet. The following amino acids were dissolved in their drinking water at a concentration of 40 mmol/l: d-Asp, n = 20 (10 + 10), d-Ser, n = 23 (10 + 13), l-serine (l-Ser), n = 20 (11 + 9). A similar time period (2 months) of oral administration has been shown to substantially increase the concentration of d-Asp in the brain^5^ in mice of the very same strain. In another study, oral administration of d-Ser to wild type CD1 mice over a period of 35 days led to a 1.5–threefold elevation of d-Ser concentration in 3 brain regions (cortex, hippocampus, forebrain)^49^. Thus we assumed that this dosing regimen would be sufficient to achieve similar results. Furthermore, this time period was considered sufficient for the maturation of BrdU labeled neuronal progenitors. The non-essential amino acid l-Ser was chosen to be fed to the control group, to account for the potential extra amino acid or calorie intake by d-Ser and d-Asp. Then, a further 9 animals receiving no supplementary amino acid were added to one of the cohorts. Since no difference was found between the results of the l-serine treated and ‘blank’ groups (Suppl. Table 1), these data were merged and referred to as ‘Control’ accordingly. Apart from the dietary supplement of amino acids, all animals were fed on standard laboratory chow, available ad libitum. One cohort of mice (Cohort 1) was used for the combined behavioural test (spatial learning) and western blotting (WB) (NMDAR expression) study (Experiment 1, n = 41), while the other cohort (Cohort 2) was used for locomotor activity and motor coordination tests, as well as for measuring changes of cell-proliferation (Experiment 2, n = 31). For both cohorts dietary supplementation lasted for approximately 7 weeks alike (Fig. 1). All animals were kept and treated according to the regulations of the ethical committee of Semmelweis University. All experiments were approved by the Ethical Committee on Animal Experimentation, and permitted by the Food Chain Safety and Animal Health Directorate of the Government Office for Pest County (Permit Number: XIV-I-001/2269–4/2012). Procedures were in harmony with the EU Council directives on laboratory animals (86/609/EEC). The study was carried out in compliance with the ARRIVE guidelines (https://arriveguidelines.org).

### Experiment 1

Effect of d-Asp and d-Ser on spatial learning and the expression of hippocampal NMDAR subunits.

We used the hidden platform version of the Morris water maze (MWM) test. A circular platform of 7 cm diameter was placed into a round pool of one meter diameter. The pool was filled with water (23 °C) mixed with harmless white Pannoncolor® gouache tempera for higher opacity. The water level above the platform (1 cm) was just enough to make it invisible to the mice, yet not deep enough for the animals to pass over the platform without touching it. Global cues around the pool included the surface texture of one of the walls (wire mesh), three paper decorations (ca. 1 m long) hanging at fixed positions, and an air filtration fixture on the ceiling. None of these cues were closer than 150 cm to the edge of the water maze.

Each individual was allowed to swim four times a day, for nine days, twice in the morning, approximately ten minutes apart, and again twice in the afternoon, ten minutes apart except on test days 46 and 49, when the morning session was replaced by a test trial. On day 43 afternoon there was only one swimming trial due to technical difficulties (Fig. 1B). Such a schedule resulted three trial types: (1) M trials: where a medium amount of time (3–4 h) elapsed after the previous trial, (2) S trials: where the preceding intertrial interval was shorter (10–15 min) and (3) L trials: where the trial was preceded by a long intertrial interval (14–16 h) (Fig. 1B). Trials lasted for two minutes or until the mouse found the platform. The test consisted of three stages.

#### Non-random start phase

In the first three days, the animals had to find the platform starting from the same point of the pool. They had a total of 2 min to search, and, if they couldn’t find their destination within that time, the experimenter helped them to the platform. There were no consistently poorly performing mice in any of the treatment groups throughout the experiment. For the occurrence of such assistance at each trial see the supplementary datafile SD1.xlsx. Once an animal found the platform, it had half a minute to survey its surroundings and then returned to its box.

#### Random start phase

Over the next three days, starts were made from four equally spaced points of the pool. None of the points coincided with the starting point of the non-random start phase. The order of the starting points was assigned by random generation on a daily basis. The platform was still in the previous position during this period. The purpose of this study was to determine whether the animals, starting from a given point, learned the route to target based on local stimuli, or else they were orientated by a mental map based on global environmental cues.

#### Random start, relocated platform phase

In the last three days of the MWM, the animals started swimming again from any of the four points used in the previous stage. In this phase the animals had to find a platform placed in a different quadrant of the basin. This method can be used to assess how flexible is the memory formed after training. More precisely: does it remain fixed to the previously learned position of the platform, and, if not, how quickly can the animals adapt to remember the changed position of the landmarks (spatial cues).

### MWM test phase

Each of the three-day swimming phases ended with a test trial. Here the mice swam in the pool for 2 min with the platform removed from the water. The purpose of this test was to verify that the animals find the platform by using previously stored spatial information (i.e. they have actually learned the position of the platform), rather than being guided by sensory experience not depending on prior learning.

All the above swimming sessions were videotaped using an overhead fixed digital camera. Latency of finding the platform was event-recorded by a stopwatch, here the videos served as permanent backup. For the test phase, the trajectory of swimming was evaluated by post-processing of video recording, using the Image J plugin AnimalTracker^50^. Distance of swimming and the time spent in different quadrants were measured.

### Elevated plus maze test

Elevated plus maze experiments were performed in a room unfamiliar to the animals. With this method, the anxiety level of mice can be indirectly assessed. Each animal was videotaped for 5 min during the study. We measured the time spent in the open arm, as compared to that spent in the closed arm of the maze. The time spent in the square area at the junction of the arms was excluded from the analysis because during their stay here, it was not possible to decide whether the animal exhibits exploratory or covert behaviour.

### Western blot analysis

Following the behavioural studies (after 7 weeks of dietary supplementation), the animals were decapitated without anaesthesia, their brains were removed and placed on ice to reduce the rate of postmortem tissue enzymatic degradation. The cortex was opened by a longitudinal incision between the two hemispheres (sagittal callosotomy), folded laterally, thus exposing the hippocampus, which was dissected in its entirety. Samples were placed in Eppendorf tubes on dry ice to inhibit protein degradation and stored in a − 80 °C freezer until further processing. The aim of the WB was to estimate changes in the total amount of NMDAR protein (by measuring the uniformly occurring GluN1 subunit) and also the selective alteration occurring in the amount of GluN2A and GluN2B subunits in whole hippocampal extracts of the differently treated animals. Since our aim was to quantitatively study the proteins of NMDARs present in the synapses, it was necessary to separate the “crude” synaptosomal fraction from the lysate.

To prepare the lysate, Syn-PER (Synaptic Protein Extraction Reagent—Thermo Fisher Scientific) lysis solution was added to the frozen samples (20 ml/g wet wt.). EDTA (Thermo Scientific ™ 0.5 M EDTA) and Halt (Halt ™ Protease Inhibitor Cocktail) were mixed with the lysis buffer in a ratio of 1: 100 each in order to completely inhibit the protein-degrading enzymes even after freezing. The tissue homogenate was prepared by sonication for 2 s at 4 C°. The homogenate was then centrifuged at 1200 × g for 10 min at 4° C, to remove cellular debris. The separated supernatant was centrifuged again at 15,000 × g, for 20 min at 4° C. The supernatant was aspirated, and the pellet (crude synaptosome fraction) was resuspended with Syn-PER (3 ml/g starting weight). Protein concentrations in the brain homogenates were determined using the BCA method^51^. Following this, all samples were diluted to an even protein concentration (2 μg/μl) by adding additional lysing solution, followed by addition of Laemmli solution at 1:1. Samples containing 10 μg of total protein (for each NMDAR subunit) were loaded onto 8% acrylamide gels and processed with BioRad Mini‑Protean III vertical electrophoresis and blotting systems. Proteins were separated by SDS‑polyacrylamide gel electrophoresis (PAGE) and electrophoretically transferred to nitrocellulose membranes. Then, the membranes were incubated with 2% non-fat dry milk (Cell Signaling) in TBS‑T buffer (0.05 M Tris‑buffered saline pH 7.4 and 0.1% Triton X) for 1 h at room temperature. The membranes were incubated with one of the following receptor antibodies (Cell Signaling, rabbit, each diluted at 1:1000): anti‑GluN1, anti‑GluN2A, anti‑GluN2B; and anti‑β‑actin (Cell Signaling, mouse, 1:100,000) primary antibody, overnight at 4 °C. After washing, HRP conjugated anti‑rabbit secondary antibody (DAKO, 1:10,000) was added to the NMDAR samples, and HRP conjugated anti‑mouse secondary antibody (DAKO, 1:20,000) to the beta‑actin samples. The blots were visualized and quantified with an enhanced chemiluminescence detection system (BioRAD) and standardized by the luminosity of the housekeeping protein beta‑actin used as a ‘loading’ control on the membranes. Since the GluN2A, GluN2B subunits both have a molecular weight of 180 kDa, these could not be assayed on the same blotting membrane together. A further technical difficulty (noted by the manufacturer) was that the GluN2A antibody produced an additional band of label at an altitude of 130 kDa (expected level of GluN1 subunit). Thus, it was also not possible to detect the GluN1 subunit simultaneously with GluN2A, on the same blotting membrane. On both types of membranes visualisation and measurement of GluN2A or GluN1 bands and those of beta actin bands were carried out separately with different exposure times to avoid overexposure of beta‑actin bands (Fig. S1-2). Furthermore, in order to achieve an ideal signal intensity in the case of GluN2B, the part of the blotting membrane containing GluN2B band was cut off and processed separately from the rest of the membrane (Fig. S1). The following exposure times were used for the different bands of the same blot: 10 s (Beta-actin); 18 s (GluN2B); 20 s (GluN1); 18 s (GluN2A). None of these procedures could have affected the ratios between the treatment groups.

### Experiment 2

 Effect of d-Asp and d-Ser on general behaviour and cell proliferation in the hippocampus.

In cohort 2, one group received d-Asp while the second received d-Ser in the drinking water for 7 weeks (Fig. 1A) at a concentration of 40 mmol/l. The third group received 40 mmol/l l-Ser with the same parameters. On the 24th day of the treatment, bromo-deoxyuridine (BrdU) was administered intraperitoneally (0.2 mg/g body weight). We chose a single large dose injection in order to saturate the cells in the given S-phase, while avoiding the incorporation of BrdU into an excessive number of neurones, potentially causing toxicity and interfering with their behaviour^52^. Behavioural testing (open field and motor coordination tests) took place on the 48th day of the treatment. The animals were killed and had their brains dissected on the 49th day of the treatment (Fig. 1A).

### Open field test

On the 48th day of treatment, the animals were positioned in the middle of a 110 cm diameter circular open field arena, with walls of 36 cm height. The animals spent 5 min in the apparatus, while the behaviour was captured by an overhead digital camera. During video analysis the following variables were recorded: latency of first ambulation, number of rearings and total distance covered. We also measured the time spent at centre of the arena.

Motor coordination test: The mice were positioned in the middle of a 40-cm-long and 2-mm-wide stainless steel rod, fixed 40 cm above the ground. The mice held onto the rod and the time until they were unable to hold themselves any more and dropped was measured. A thick layer of soft tissue paper was placed under the rod to prevent the mice from hurting themselves when falling down. The latency of falling was expressed as the mean of two consecutive trials for every individual, and compared between the experimental groups.

### Tissue sampling and histology

On day 49 of Experiment 2, the animals were anaesthetised with a mixture of ketamine (460 mg/kg b.w.) and xylazine (80 mg/kg b.w.), and transcardially perfused with 4% paraformaldehyde in 0.1 M phosphate buffer. Whole brains were dissected and postfixed overnight at 4 ^o^C in the same fixative. The brains were then transferred to a 30% sucrose solution in 0.01 M phosphate buffer (pH 7.4) in saline (PBS) for cryoprotection. Brains then were dissected into 6 series of 40 µm thick coronal sections using a freezing microtome (Leica Frigomobile).

On one series of free‐floating sections horseradish peroxidase catalyzed diaminobenzidine (3,3′‐diaminobenzidine tetrahydrochloride)—DAB reaction was used to visualize the expression of doublecortin (DCX) a marker for immature, migrating neurones, whereas in another series of sections from the same brains, immunolabelling against NeuN and BrdU was visualized using multiple immunofluorescence. Sections were washed in 0.01 M PBS and then immersed in 3 N HCl for 20 min, washed again in 0.01 M PBS, and blocked in 1% bovine serum albumin (for NeuN) or donkey serum (for DCX) for 30 min. Primary antibodies were then applied: sheep monoclonal anti‐BrdU (Novus Biologicals), diluted at 1:200; rabbit polyclonal anti‐NeuN, diluted at 1:10,000; goat polyclonal anti‐DCX (Santa Cruz Biotechnology sc-8066), diluted at 1:300 for 48 h at 4 °C. After three washes in 0.01 M PBS, sections were incubated with secondary antibodies for 2 h at room temperature: for BrdU, DyLight550 labelled donkey anti‐sheep IgG (Invitrogen, Carlsbad, CA); for NeuN, Alexa 488‐conjugated donkey anti‐rabbit IgG (Invitrogen). For the DCX, the secondary antibody was biotinylated donkey anti‐goat IgG (Vector, Burlingame, CA) diluted at at 1:200. Excess antibodies were washed off with 0.01 M PBS. For DAB visualization of DCX, sections were pre-treated in 1% H_2_O_2_ in PBS (pH 7.4) for 15 min, rinsed four times in PBS, incubated in avidin‐biotinylated horseradish peroxidase complex (ABC; diluted at 1:200 in PBS, pH7.4; Vector, Burlingame, CA) overnight at 4 °C, and then rinsed four times in PBS. The sections were then incubated for 10 min in Tris buffer (0.05 M Tris‐HCl, pH 8.0) containing 0.015% DAB and 0.25% nickel‐ammonium sulphate. After 10 min, 1% H_2_O_2_ was added at a final dilution of 0.01%. The reaction was visually controlled and stopped by rinsing first in Tris buffer (pH 8.0) then in PBS (three times) as soon as DCX labelling became evident. Those sections with fluorescent label against NeuN and BrdU were mounted onto glass slides and coverslipped using a 1:1 mixture of PB and glycerol, whereas DAB‐labelled sections were mounted on gelatine/chromalum‐subbed slides, air dried, dehydrated through graded alcohols, cleared with xylene, and finally coverslipped with DePeX (Fluka/Sigma‐Aldrich, Steinheim, Germany).

### Quantitative analysis

All doubly labeled BrdU + / NeuN + neurones were counted manually under an Olympus BX51 fluorescent microscope using the method of Eisch et al.^53^ in every sixth section of the hippocampal tissue that included the DG. Since the caudalmost part of the hippocampus was often separated from the rest of the section and occasionally lost during the immunohistochemistry procedure, this part was systematically omitted from the analysis. Labelled cells in the rostral eight 40 µm sections (240 µm apart) were counted, spanning the rostrocaudal length of 1920 µm. This constitutes approximately 70% of the total rostrocaudal extent of the DG, based on the Allen Mouse Brain Atlas^54^. The total number of the double labelled (BrdU + and NeuN +) cells within this representative volume of the DG was calculated by the sum of such cells measured at the 8 representative sectional levels, multiplied by six.

From every brain, three reference sections were selected at the positions − 1.155, − 1.955and − 2.255 mm according to bregma (see the Allen Mouse Brain Atlas;^54^), and every DCX labelled cell was counted bilaterally using the same Olympus BX51 microscope in transmitted light mode. The number of DCX cells were counted separately in the granular cell layer and the subgranular zone of the DG. The cells were counted manually under the microscope, adjusting the focus for an optimal detection of cells throughout the thickness of the section. The total length of the subgranular zone of the DG was measured on photomicrographs using the ImageJ software^55^. The number of cells in both the subgranular and granular layer were normalized to the length of the subgranular zone in millimetres, in order to make it comparable across the different brains.

### Statistical analysis

Labels on the slides containing the brain slices and names of the video files were recoded to avoid bias in the analysis of cell counting and behaviour. The codes were broken after the analysis had been finished.

The two control groups (l-Ser and no amino acid) were merged based on Welch two sample t-tests, since there was no significant difference between these in any of the observed variables (Table S1). Data are given as means of the individuals within a group ± standard error of the mean.

In Experiment 1 repeated measures general linear models were used check the effect of DAA treatment or the time elapsed from the previous swimming. The model contained latency as a dependent variable, DAA treatment and trial types (S, M and L) as explanatory factors and their interactions. Tukey post-hoc tests were used for pairwise comparison between d-Asp and d-Ser consuming groups and the controls. Data from separate phases of the MWM were fed into separate models. For test trials, Welch paired sample t-tests were used to compare the time spent in the target zone (where the platform was placed in the previous learning sessions) with the time spent in other (not target) zones. In the case of the Elevated Plus Maze Test, a general linear model with Tukey post hoc tests was used to compare the d-Asp and d-Ser consuming groups with the controls. Model formula: Time spent in open arm (as dependent variable), DAA treatment (as explanatory factor). General linear models were used to compare the normalised NMDAR subunit values. Model formula: NMDAR subunit value (as dependent variable), DAA treatment (as explanatory factor) and membrane ID (as a random factor).

In Experiment 2 general linear models (cell numbers and behavioural measures as dependent variables, and the DAA treatment as the explanatory factor) with Tukey post-hoc tests were used to compare the d-Asp and d-Ser consuming groups with the controls. Data are given as averages of the individuals within a group ± standard error of the mean.

## Results

The 7-week consumption of DAA-enriched drinking water did not result in any apparent phenomenological difference with the control animals (general condition, appearance or behaviour). The body weight did not differ among the groups at the end of the behavioural experiments either in experiment 1 (F_2,28_ = 1.5, *P* = 0.241) or in experiment 2 (F_2,37_ = 1.33, *P* = 0.280).

### Behavioural tests

**Figure 2:**
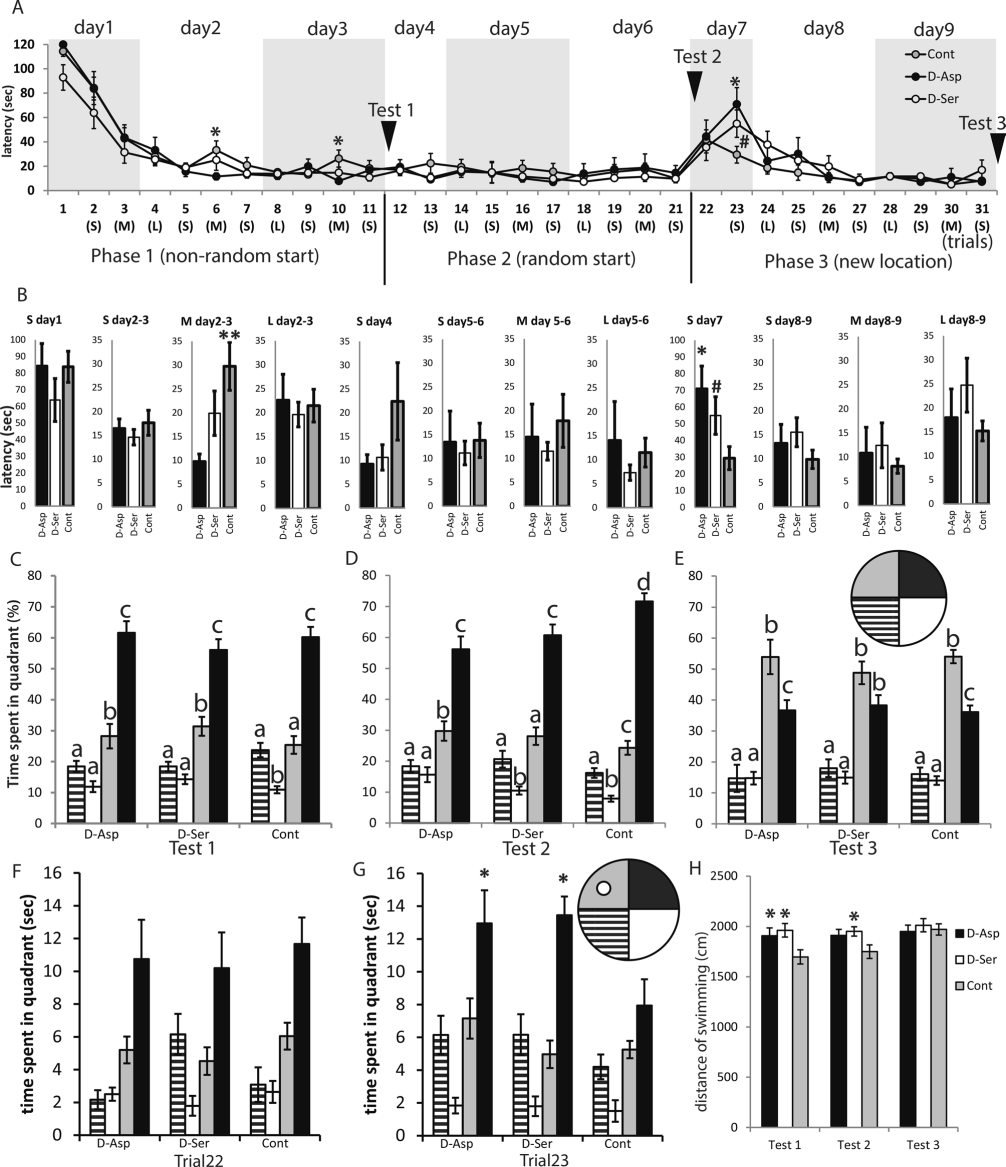
(**A**) Latency of finding the platform in the Morris water-maze test. The training consisted of three distinct phases over the span of 9 days: (1) When the mice were put into the maze consistently at the same point of the basin to start their exploration (non-random start), (2) when their position was chosen randomly around the edge of the maze (random start), and (3) when the platform was relocated to another quadrant (new location). In phase 1, d-Asp treated mice reached the platform faster than control individuals when a medium amount of time (3–4 h) passed after the previous trial (M) but there was no such difference in the latency either in case of a short (S) (10–15 min), or a long (L) (14–16 h) intertrial period. In phase 2, the random starting position did not affect the performance of the individuals in any of the treatment groups. In phase 3, both d-Ser and d-Asp mice were slower than controls to reach the platform when its location was changed. Asterisk (*) represents significant difference between d-Asp and Control (*P* < 0.05; Tukey post-hoc test). Hash (#) represents significant difference between d-Ser and Control (*P* = 0.064; Tukey post-hoc test). (**B**) Diagrams representing the mean latencies of S, M and L trials (as defined in Fig. 1A) in the acquisition period (days 1, 4, 7) or consolidation period (combining days 2–3, 5–6, 8–9). The advantage of the d-Asp individuals in learning the location of the platform was only detectable during phase 1 of learning, in the consolidation period (on days 2–3 of training), in those trials that followed the previous trial after 180–240 min (M). When the platform was relocated to a different quadrant of the water maze, both d-Ser and d-Asp individuals were slower to adapt to the change, however they differed from the control animals only on day 7, at the second trial (10 min after the first trial with the new location). A transient delay of reversal learning with both d-Ser and d-Asp was observed on day 7 (S). *P < 0.05, **P < 0.01, #*P* = 0.064. (**C**–**E**) To show that the animals indeed learn the position of the platform (black quadrant) and not using any direct cues to detect it, we tested every animal without the platform after all the three phases of the trainings. The different letters above the columns denote significant difference between the time spent in the quadrants within one treatment group. All mice learned the spatial location of the platform, and spent more time in the appropriate quadrant even with the platform removed. During the reversal (E) d-Asp treated and control animals preferred the new location of the platform (grey quadrant), although the previous location remained their second preferred choice. d-Ser treated mice also tended to prefer the new location over the previous one, however their preference just failed to reach the level of statistical significance (t = 2.47, *P* = 0.082). (**F**–**G**) During the second (but not the first, F) trial of the reversal learning (G), both d-Asp and d-Ser individuals spent longer time in the quadrant of previously learned platform location when compared with control mice (d-Asp: t = 2.13, *P* = 0.039; d-Ser: t = 2.54, *P* = 0.015). Gray quadrant: new target zone, black quadrant: previous target zone. *P < 0.05 compared to control. (**H**) Both d-Asp (t = 2.13, *P* = 0.040) and d-Ser (t = 2.90, *P* = 0.006) mice covered more distance while swimming than the control animals, in the first platformless test (F_2,38_ = , *P* = 0.014). However, during the second test (F_2,38_ = 3.21, *P* = 0.05) session, only d-Ser differed from the controls (t = 2.38, *P* = 0.023). There was no significant difference during the third test session. *P < 0.05 compared to control.

All groups learned to find the hidden platform in the Morris water-maze test at approximately the same pace in the first phase of the MWM experiment (F_8, 1138_ = 0.70, *P* = 0.688). Differences were only apparent in the second and third days of the training (F_8, 319_ = 2.39, *P* = 0.016) where there was a significant interaction between the trial type (S, M, L) and DAA treatment (F_4, 281_ = 2.40, *P* = 0.049). There was no such effect in case of the second (non-random start) phase (F_8,360_ = 0.53, *P* = 0.832). The only difference in latency between the groups was that on the second and third day of training the performance of the control group somewhat declined. d-Asp treated mice showed no such deterioration of performance, representing a significant difference between d-Asp and control animals (Fig. 2A). This difference was restricted to the trials executed 180–240 min after the previous trials (Fig. 2B, F2,38 = 4.384, *P* = 0.019). The performance of d-Ser treated animals fell between those of d-Asp and Control and did not differ significantly from either (t = 1.56, *P* = 0.126Fig. 2B). When the starting location of the mice was changed to random on the fourth day of training, all of the three groups performed equally well (F_2,38_ = 0.34, *P* = 0.715, Fig. 2A) and similarly to the previous session, suggesting that they already learned the global spatial cues in the first phase (with a fixed starting point), instead of using local cues. However, when the location of the platform was changed on day 7, the performance of all groups dropped. Both d-Ser and d-Asp individuals re-learned the new position slower than the controls (F_2,38_ = 4.488, *P* = 0.018, Fig. 2A), the difference in the latency in trial 23 was only significant with d-Asp (t = 2.87, *P* = 0.007), whereas only a trend was evident with d-Ser (t = 1.91, *P* = 0.06, Fig. 2B). All groups learned the location of the platform within the given time, as shown by the test sessions without the platforms at the end of every phase (Fig. 2C-E). All groups spent more time in the quadrant with the previously learned platform than in any of the other quadrants, and there was no difference between the treatment groups (F_6,114_ = 1.40, *P* = 0.221). During the reversal test trial (Fig. 2E), all animals preferred the new location of the platform, although the previous location remained their second preferred choice. There was no difference between the experimental groups either in the preference for the new platform location (F_2,38_ = 0.72, *P* = 0.492), or in the preference for the previous (original) platform location (F_2,38_ = 0.17, *P* = 0.842). Nor was there any difference between the groups (Fig. 2F, F2,38 = 0.16, *P* = 0.855) at the first trial of the reversal training (day 7, trial 22): the mice kept searching the platform at its previous location. However, during the second trial of the reversal training (day 7, trial 23) both d-Asp and d-Ser individuals spent more time searching the platform at the previous location than did the control mice (Fig. 2G, F2,38 = 3.94, *P* = 0.028). This was most prominent in the first 30 s of the trial, a time interval during which an average control mouse already found the platform. This phenomenon likely represents protracted perseveration in d-AA consuming mice.

**Figure 3:**
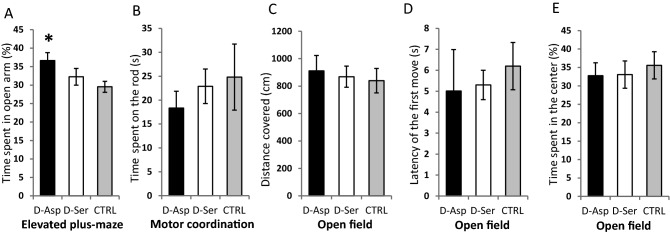
(**A**) d-Asp treated mice spent more time in the open arm of the elevated plus-maze than control animals (t = 2.61, *P* = 0.014). *P < 0.05. (**B**) Neither of the experimental treatment groups differed from the control when tested for motor coordination by a rod grip test (F_2,27_ = 1.27, *P* = 0.297). (**C**) There was no significant difference in the locomotor activity of the treatment groups in an open field test (F_2,28_ = 1,98, *P* = 0.157). (**D**) There was no difference between the groups regarding the latency of the first movement after placing the mouse in the middle of arena (F_2,28_ = 0.901, *P* = 0.418). (**E**) There was no difference between the treatment groups in the time spent at the center of the open-field arena (F_2,28_ = 0.18, *P* = 0.838).

In order to check whether the observed differences in the spatial learning task could, in part, be attributed to differences in the physical condition and activity of different groups, we first measured the total length of swimming in all three platformless tests (Fig. 2H). In test 1, both d-Asp and d-Ser treated mice swam more than the controls (F_2,38_ = 4.786, *P* = 0.014), but the controls gradually caught up with the d-AA treated animals by test 3 (F_2,38_ = 0.215, *P* = 0.807). A reduced level of anxiety was observed in the elevated plus maze test (F_2,38_ = 3.292, *P* = 0.048) in the case of d-Asp (t = 2.57, *P* = 0.014) – but not d-Ser (t = 1.06, *P* = 0.296) – mice (Fig. 3A). Furthermore, neither the d-Asp nor the d-Ser mice showed any difference from controls when tested on a motor coordination test (their ability to cling on to a stainless steel rod) (Fig. 3B, F2,28 = 1.27, *P* = 0.297). When tested in a circular open-field arena, there was no difference in locomotor activity between the groups (Fig. 3C), (F_2,28_ = 1,98, *P* = 0.157). There was no difference between the groups regarding the latency of the first movement (duration of freezing, Fig. 3D) after placing the mouse in the middle of arena (F_2,28_ = 0.901, *P* = 0.418). In the open field test all mice spent less time at the center of the arena; no difference was observed between the groups regarding this variable. (Fig. 3E, F2,28 = 0.18, *P* = 0.838). The frequency of escape behaviour (rearing at the wall) did not differ among the groups either (F_2,28_ = 0.708, *P* = 0.501) (not shown).

### Western blot

**Figure 4:**
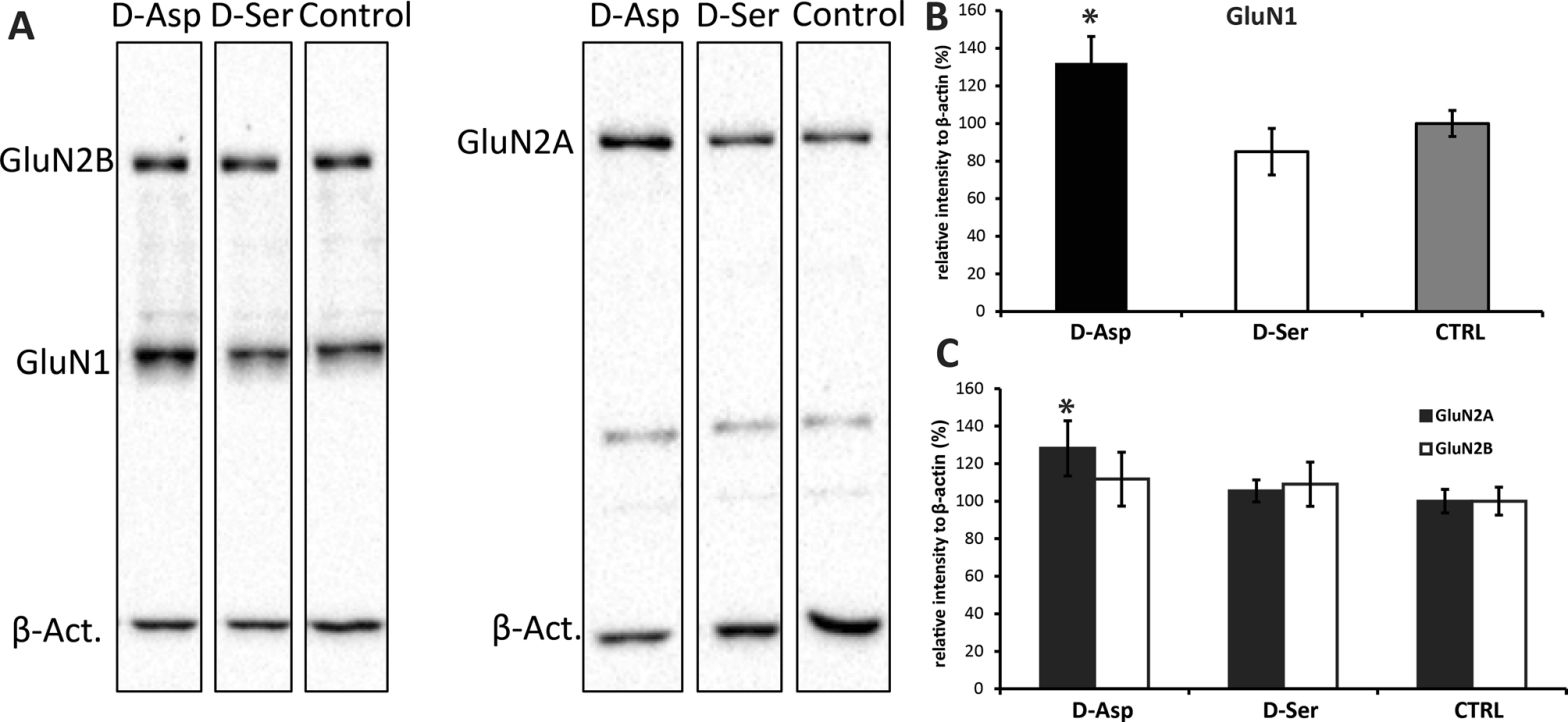
(**A**) Representative blotting membranes showing the position and density of the subunits of NMDARs in the crude synaptosome fraction of hippocampal tissue extracts. The GluN2A and GluN2B subunits were run on different membranes for technical reasons. The bands corresponding to respective GluN subunits and to beta-actin were developed using different exposure times prior to scanning. Region of the blot containing GluN2B subunits has been cut off and processed separately. For full length blots see supplementary Figures S1 and S2. (**B**) The expression of GluN1 subunit was significantly elevated in the hippocampus of d-Asp consuming mice as compared to controls (*t = 2.05, *P* = 0.048). (**C**) d-Asp mice also showed greater expression of the GluN2A subunit as compared to controls (*t = 2.32, *P* = 0.026), but there was no significant difference in the expression of GluN2B.

An elevated expression of GluN1 subunit of the NMDAR was observed (F_2,36_ = 4.05, *P* = 0.026) in the crude synaptosome fraction of the hippocampus in d-Asp (but not d-Ser) consuming mice, compared with controls (Fig. 4A, B). Such change was accompanied by an increase in the expression of GluN2A (F_2,36_ = 2.745, *P* = 0.021), but not GluN2B (F_2,36_ = 0.367, *P* = 0.6951), evident only in the d-Asp group (Fig. 4A, C).

### Neurogenesis in the dentate gyrus

**Figure 5:**
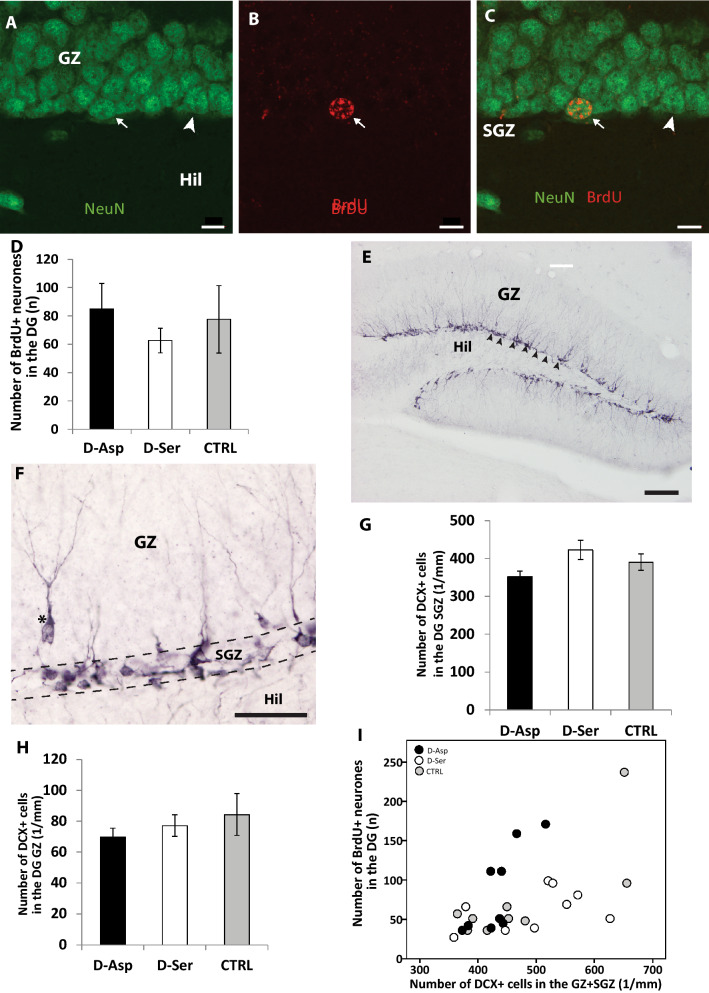
(**A**–**C**) Confocal laser scanning photomicrographs of the granular layer of the dentate gyrus immunolabelled against NeuN (green) and BrdU (red). A-NeuN labelled granule cells visualized using the green channel, (**B**) A BrdU containing cell nucleus in the same region (red channel), (**C**) Combined images of A and B to demonstrate that the BrdU + nucleus belongs to a NeuN + neuron. Arrow: BrdU + /NeuN + doubly labelled neurone, Arrowhead: NeuN + neurone, Scale bar: 10 µm. Abbreviations: GZ-granule cell zone, Hil – hilus, SGZ – subgranular zone. (**D**) Calculated number of newly generated (BrdU +) cells of verified neuronal character (colocalizing with NeuN +) in the given reference volume of dentate gyrus. The treatments had no effect on the number of BrdU + neurones. (F_2,24_ = 0.445, *P* = 0.646). (**E**) Photomicrograph of the dentate gyrus immunolabelled for doublecortin (DCX). The somata and processes of migrating precursor cells are visible at the granular/subgranular border. Arrowheads mark the cells lining up in the subgranular zone, GZ-granule cell zone, Hil-hilus. Scale bar: 200 µm. (**F**) DCX + cells tends to accumulate at the subgranular proliferative zone (SGZ) of the dentate gyrus, however some of the newly proliferated cells (asterisk) already started to migrate into the granular zone (GZ). Hil-hilus. Scale bar: 50 µm. (**G**–**H**) Number of DCX + cells measured in a reference volume of the dentate gyrus of mice treated with d-amino acids. The chronic consumption of neither d-Asp (Tukey: *P* = 0.419) nor d-Ser (Tukey: *P* = 0.526) facilitated the production of DCX + neurones, compared to controls, in the subgranular (G) (F_2,28_ = 1.482, *P* = 0.220) and granular (H) (F_2,28_ = 0.558, *P* = 0.578) zone. (**I**) The number of BrdU + neurones and DCX + cells show positive correlation in the dentate gyrus (R^2^ = 0.536, n = 27, *P* = 0.004).

All of the mature neurones in the subgranular zone of DG (Fig. 5A) that had undergone division after the BrdU injection (Fig. 5B), migrated into, and settled in the granular zone (Fig. 5C) 25 days later (when the histological samples were taken). No difference was observed among the experimental groups in the number of BrdU expressing mature (NeuN +) neurones in the DG (Fig. 5D, F2,24 = 0.445, *P* = 0.646). This suggests that neither of the d-amino acids influenced the survival or maturation of the neuronal progenitor cells. While the nuclei of mature neurones were clearly visible, those nuclei that contained BrdU but did not express NeuN were difficult to identify in the tissue, and therefore were not quantified. The newly divided neuronal progenitors expressing DCX (Fig. 5E) were mostly located in the subgranular zone, with a few already starting their migration into the granule cell layer (Fig. 5F). There was no difference between the experimental groups in the number of DCX cells either in the subgranular (Fig. 5G, F2,28 = 1.482, *P* = 0.220) or in the granular layer (Fig. 5H, F2,28 = 0.558, *P* = 0.578). Despite that BrdU and DCX were measured on different sections and those sections had undergone different immunohistochemical procedures, the number of BrdU + neurones and the total number of DCX (GZ + SGZ) in the DG were strongly correlated (Fig. 5I, *R*^2^ = 0.536, n = 27, *P* = 0.004).

## Discussion

### Interpretation of behavioural findings

One possible reason why we were able to detect subtle changes of spatial learning in mice could be that these animals were trained over a relatively long period, based on local and global cues in the initial (non-random start) phase, which resulted in a stronger memory trace. Apparently, in our hand, a detailed analysis of the behaviour in selective post-training periods, including within-day session data, revealed the effect of d-Asp on the consolidation of memory more precisely than what has been reported in previous studies. In the present study, a cognitive advantage (though rather limited in extent) of the d-Asp consuming individuals, as compared to controls, was clearly manifested. The greatest difference between control and d-Asp treated mice was measured in the intermediate post-training period (3–4 h). In fact, d-Asp treatment seems to abolish the latency rebound episodes occurring in the control animals (also evident, though not necessarily discussed, in other studies – e.g. in Errico et al.^5^), rather than accelerating acquisition itself. Overall, the observed effect is far less robust than the cognitive enhancement reported for rats^6^. Such memory rebounds are often obscured by the fact that, in most experiments reported, the results are presented as daily averages^17,56^. Our observation can be interpreted also in terms of memory reconsolidation. Transient destabilization and reconsolidation of memory during retrieval are key phenomena^57,58^ in memory formation, also allowing for updating and plasticity. Thus, the principal effect of d-Asp could be rendering the memory trace more resistant to destabilization. Indeed, an important function of memory destabilization and reconsolidation following retrieval is to increase the persistence or precision of the memory trace^59–62^. Prolonged training during Phase 1 with several bouts of reconsolidation likely formed a strong enough memory trace to mask the subtle effects of d-Asp in Phase 2. Apparently, the effect of d-Asp was only prominent in the case of intermediate intertrial intervals (M). The likely reason why no latency rebounds (prone to d-Asp action) occurred elsewhere is as follows. After the short intertrial intervals (S) the animals may have used short term memory, unaffected by destabilization, whereas after long intertrial intervals (L) memory had plenty of time anyway to fully reconsolidate into long term memory.

On the other hand, elimination of latency rebound episodes can also lead to diminished plasticity and perseverance. That this might indeed be the case, is further supported by the protracted reversal learning observed by us here in d-Asp (and, less evidently, in d-Ser) treated mice.

Curiously, there are very few studies on the behavioural effects of protracted elevation of endogenous d-Ser (see Peyrovian et al. 2019)^63^. As evidenced by reports on acute administration of the amino acid, d-Ser acts as an enhancer of spatial memory and cognitive flexibility^45,46^. Compared with these experiments, in the present study d-Ser was administered chronically for weeks, which might have caused long term changes in the d-Ser production or metabolism via compensatory or habituation processes. By contrast with d-Asp, d-Ser did not significantly alter the performance curve in finding the position of the hidden platform. However, it cannot be excluded that d-Ser did prolong the initial phase of reversal learning without abolishing learning itself (just like d-Asp). Notably, the latter could be interpreted either as diminished plasticity or as a more persistent memory trace.

### Interpretation of NMDAR responses

The time period (3–4 h post-training), within which the d-Asp supplemented animals show a distinct enhancement over the control animals in performance (in remembering the position of the hidden platfom) corresponds to the first wave of de novo protein synthesis, required for L-LTP^64^. By reinforcing specific synaptic connections, L-LTP makes the responses of memory circuits more selective to given stimuli^65^. Reconsolidation is also known to depend on NMDARs: inactivation of NMDARs disrupts reactivated spatial memory^56^. In the present study, we observed a significant enhancement of the GluN1 subunit protein in the hippocampus of d-Asp treated mice as compared to control mice, pointing to an overall increase (upregulation) of NMDARs. As for the GluN2, our WB data suggest an elevated expression of GluN2A in d-Asp treated animals, potentially raising the probability of LTP formation in most (but not all) hippocampal synapses. Similar elevation of GluN1 and GluN2A was reported in the midbrain of DDO^−/−^ mice^66^ but not in the hippocampus of d-Asp exposed mice^21^. As for the latter study, a possible reason for the apparent contradiction is that the cited authors used whole tissue homogenate, while we used crude synaptosome fraction for the determination of NMDAR subunits. As pointed out by Pistillo et al.^67^, the values based on crude synaptosome fraction might better represent the mature and plasma membrane associated contingent of receptor proteins.

The upregulation of the GluN2A, also observed in the present study, is thought to contribute to stabilization of the memory trace^68^ (manifested both in perseveration during reversal and in more stable performance during Phase 1 in the d-Asp mice). Apparently, in our hand, elevation of GluN2A was not accompanied by a significant decrease of GluN2B, presumably because these two subtypes are not mutually exclusive. In the hippocampus, GluN2B subtypes tend to occur mostly in triheteromeric complexes^27^. Stable expression of GluN2B subunits (also supported by LTP-dependent recruitment) may protract the decay of LTP, thus helping sustain the existing memory^27^. We observed this phenomenon here in the form of slower reversal learning (perseverance) in the d-Asp and d-Ser treated animals. One established mechanism of forgetting, LTP reversal^69^, was found to be enhanced by elevated GluN2A, or GluN2A/GluN2B ratio^70^. Since in our study GluN2A was elevated (only in case of d-Asp treatment), yet reversal learning was slower, a direct involvement of LTP reversal is unlikely.

Apparently, the observed anxiolytic effect (potentially attenuating distraction of the animals in searching of the target) in the d-Asp treated group (as measured by the elevated plus maze test) would weaken the suggestion of a genuine memory stabilizing action. However, had this anxiolytic effect been the sole reason for improved learning, it would have been evident in all post-training phases, and not restricted to a specific (intermediate, M) intertrial phase, as in the present study. In any case, our currently available data fail to explain the observed reduction of anxiety level. Since the effect of d-Asp is not specific for the regions instrumental in spatial learning, the causal factors of reduced anxiety are likely associated with other limbic brain regions not investigated here.

By contrast with d-Asp, we did not find any changes in the expression of NMDAR subunit proteins in the animals treated with d-Ser. Hippocampal LTP was found to be absent in serine racemase (SR)^−/−^ mice (lacking d-Ser) but it could be restored by bath application of d-Ser^47^. SR^−/−^ mice also showed a deficit in spatial reference memory^47^. Notably, reversal of this deficit by administration of d-Ser was not attempted in the latter study, which makes a direct comparison with our results difficult. In apparent contrast with the present study, d-Ser was found to enhance reversal learning in MWM^34^, and this effect was sensitive to GluN2B antagonist^46^. One important difference between the conditions of the latter study and those of ours is that the cited authors employed short term pre-training injections of d-Ser, whereas our animals were chronically treated with this amino acid in the drinking water, which is known to lead to an elevated level of d-Ser over a longer period^49^.

### Interpretation of neurogenesis data

As a co-agonist of NMDAR, d-Asp has been suggested to stimulate adult neurogenesis in the dentate gyrus (GD), whereas the deficiency of d-Asp reduced the survival of nerve cells^31^. Kim and colleagues have supposed that the glutamic-oxaloacetic transaminase 1-like 1 (GOT 1l1), an enzyme that promotes the production and survival of neurones in the adult mouse brain, might be the long-sought enzyme to produce d-Asp^31^. However, later on Tanaka-Hayashi et al. (2015)^32^ showed that GOT 1l1 had no significant aspartate racemase activity. Therefore, its neurogenetic effect is most likely independent of d-Asp. By and large, our results do not support the assumption that altered adult neurogenesis in the hippocampal formation would underlie the observed changes of spatial learning and the shift in NMDAR subunit composition. No change was detected in the dentate gyrus, in the number of proliferating (DCX +) cells, or in newly born BrdU + /NeuN + neurons, either in the d-Asp-supplemented or in the d-Ser-supplemented group, compared to the control groups. Though the total number of new neurones in the DG (Fig. 5) seems to be rather low, it is comparable to the numbers found earlier by Mandyam et al. (2007)^52^ 240–720 h after a single dose of BrdU injection. Notably, the number of BrdU + /NeuN + cells did correlate well with the number of DCX + cells in the same individual. Considering the agreement with literature data and the strong correlation between the two independent measurements of cell proliferation (DCX and BrdU-NeuN) in the present study, it seems likely that our methods gave a reliable measure (’snapshot’) of the neuronal proliferation events over the given time period.

At variance with our observation, a substantial increase of newly born DG granule cells by d-Ser was reported in adult mice^48^, albeit with notable differences in experimental conditions with those applied by us. In particular, d-Ser was given i.p. over a short period, later than the time of BrdU injection (emulating a phasic effect), whereas, in our case, the animals had received d-Ser as a drinking supplement over a long period before and after the administration of BrdU (emulating a tonic effect of the DAA).

d-Asp has been implicated in the promotion of neurogenesis^13,39^ in particular via an NMDAR-related mechanism (notably, the GluN2B subunit may mediate both survival and death signals, see the instructive paper by Martel et al.)^71^. Yet it appears that, at least under the conditions applied, the learning-associated and receptor-based changes reported here cannot be ascribed to an enhanced proliferation and recruitment of new neurones. Rather, the principal factors underlying the observed memory enhancement are likely associated with an overall upregulation of NMDAR, as well as a reorganization of subunit assemblies of NMDARs in existing hippocampal/dentate neurons.

## Conclusion

This study is the first to investigate the correlated effect of d-Asp, compared to similar effects of d-Ser, on spatial learning and hippocampal neurogenesis in healthy adult mice. Following chronic consumption of d-Asp, an enhanced resistance of the memory trace to transient destabilization at a specific time period (3–4 h post-training) was found in a Morris water maze task. The same treatment also caused protraction of reversal learning, possibly due to a reconsolidation-related common mechanism manifested in perseverance. No similar effect on learning was observed following the chronic consumption of d-Ser, except for a moderate trend in reversal learning. Underlying the behavioural responses, a concomitant elevation of the expression of GluN1 (representing total NMDARs), and the elevation of GluN2A were observed in d-Asp (but not d-Ser) supplemented animals. Upregulation of NMDARs is expected to promote the onset of LTP, whereas the upregulation of the GluN2A will likely slow down the decay kinetics of the NMDA-EPSC, thereby helping to stabilize the memory trace (also manifested in perseveration). None of the learning-related changes were accompanied by alterations of hippocampal neurogenesis either in d-Asp supplemented or in d-Ser supplemented animals.

## Supplementary Information

Supplementary Information 1.

Supplementary Information 2.
