# Supplementary material for: d-Aspartate consumption selectively promotes intermediate-term spatial memory and the expression of hippocampal NMDA receptor subunits
Source: Sci Rep. 2021 Mar 17;11:6166. doi: 10.1038/s41598-021-85360-w (PMC7969773; doi:10.1038/s41598-021-85360-w)
Supplement: Supplementary file 1 — Supplementary Information 1. [file 41598_2021_85360_MOESM1_ESM.docx]

D-Aspartate consumption selectively promotes intermediate-term spatial memory and the expression of hippocampal NMDA receptor subunits.

Gergely Zachar*^#^, Róbert Kemecsei*, Szilvia Márta Papp, Katalin Wéber, Tamás Kisparti, Teadora Tyler, Gábor Gáspár, Tamás Balázsa, András Csillag.

Department of Anatomy, Histology and Embryology, Faculty of Medicine, Semmelweis University, Budapest, Hungary

*Gergely Zachar and Róbert Kemecsei should be considered joint first authors.

^#^corresponding author

Address: 58 Tűzoltó u., Budapest, 1094, Hungary

Phone: +36-1-459-1500 ext.53714

Fax: +36-1-215-5158

e-mail: [gzachar@gmail.com](mailto:gzachar@gmail.com)

Table S1

| Variables compared between the two type of controls | Welch t | d.f. | p |
| --- | --- | --- | --- |
| latency of finding the platform in the MWM at trial 6: | 1.65 | 12.72 | 0.124 |
| latency of finding the platform in the MWM at trial 10: | 0.17 | 13.85 | 0.864 |
| latency of finding the platform in the MWM at trial 23: | 0.11 | 13.12 | 0.914 |
| distance of swimming in the MWM at Test 1: | 1.17 | 15.98 | 0.261 |
| distance of swimming in the MWM at Test 1: | 0.39 | 10.67 | 0.701 |
| distance of swimming in the MWM at Test 1: | 1.68 | 14.96 | 0.114 |
| staying in the open arm in the elevated plus maze: | 1.50 | 15.89 | 0.153 |
| relative intensity of GluN1 subunit: | 0.57 | 15.82 | 0.577 |
| relative intensity of GluN2A subunit: | 2.11 | 15.10 | 0.052 |
| relative intensity of GluN2B subunit: | 1.11 | 12.57 | 0.287 |
| body weight: | 1.33 | 16 | 0.203 |

**Supplementary figures:**

**Figure S1**

**
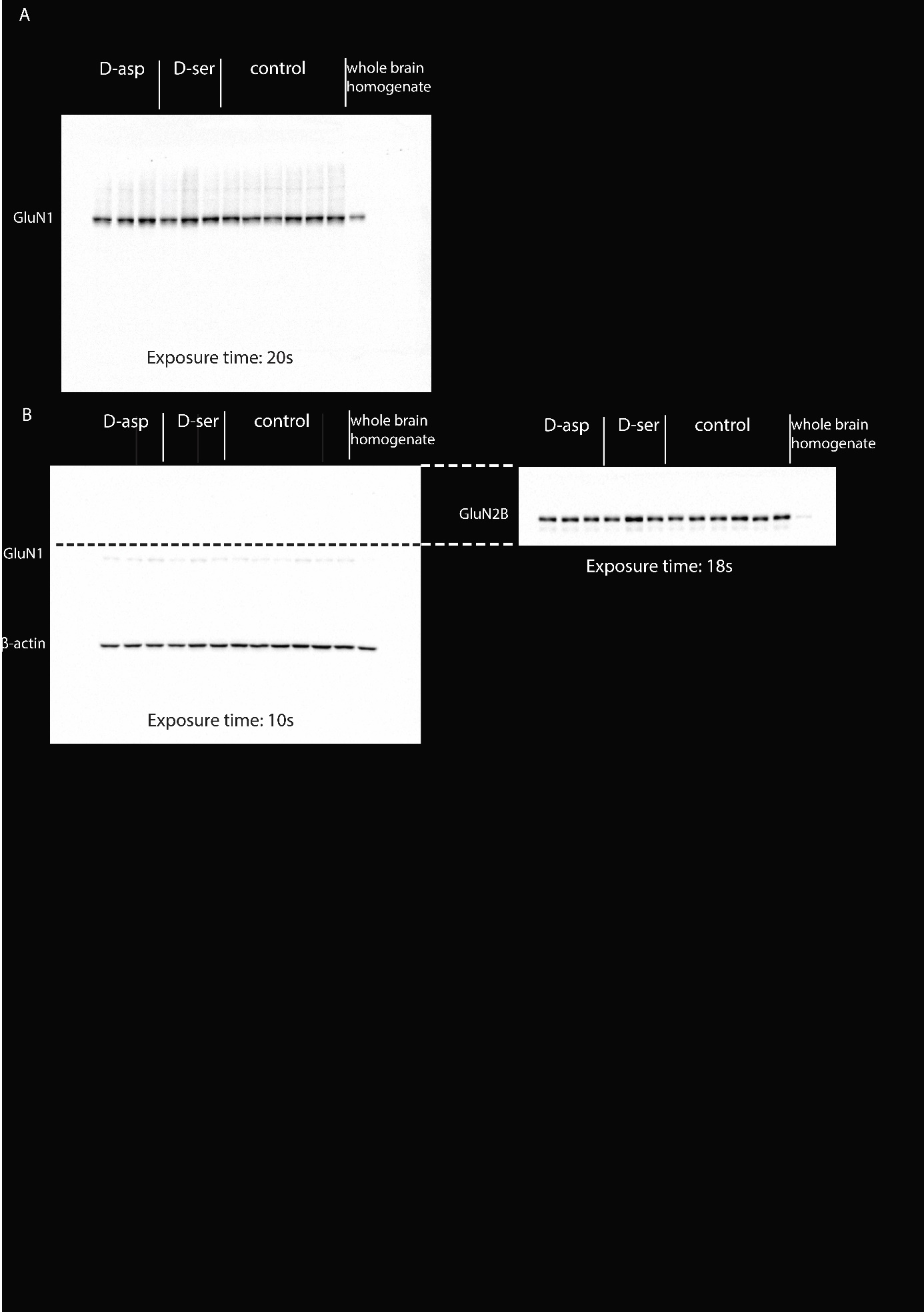
**

**Figure S2**

**
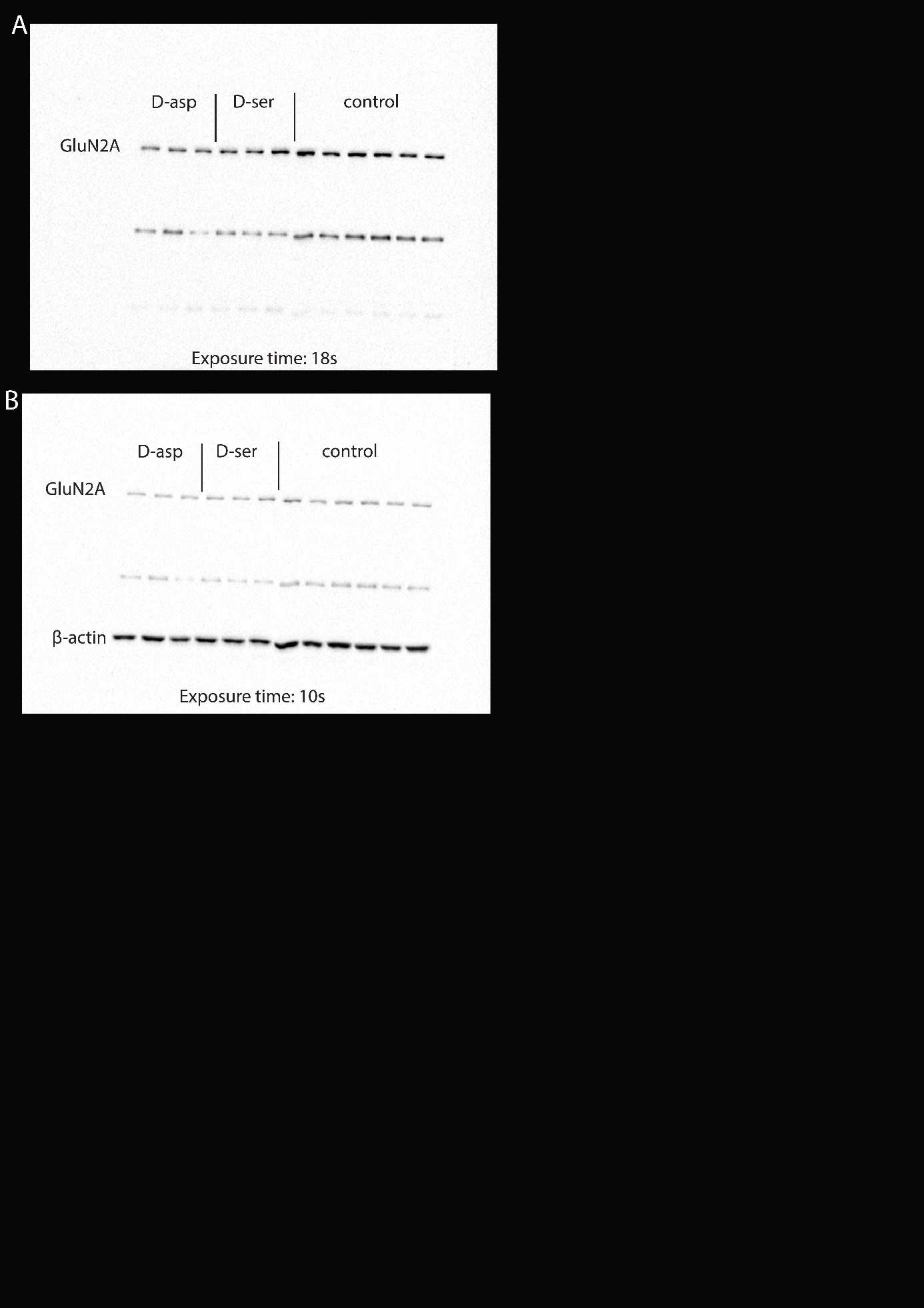
**

**Supplementary figure legends:**

**Figure S1**

Different exposures of full-length blots corresponding to the cropped images of Fig 4. Exposure for the measurement of GluN1 subunit (A). Beta-actin was labelled, visualised and measured afterwards on the same blot (B), meanwhile the region of the blot that contained the GluN2B subunit was cut off and processed separately (dashed line).

**Figure S2**

Different exposures of full-length blots corresponding to the cropped images of Fig 4. Exposure for the measurement of GluN2A subunit (A). Beta-actin was labelled, visualised and measured afterwards on the same blot (B).
